# A cross-sectional study of knowledge of sex partner serostatus among high-risk Peruvian men who have sex with men and transgender women: implications for HIV prevention

**DOI:** 10.1186/1471-2458-13-181

**Published:** 2013-02-28

**Authors:** Sharita Nagaraj, Eddy R Segura, Jesus Peinado, Kelika A Konda, Patricia Segura, Martin Casapia, Abner Ortiz, Silvia M Montano, Jesse L Clark, Jorge Sanchez, Javier R Lama

**Affiliations:** 1 South American Program in HIV Prevention Research, University of California, Los Angeles, USA; 2 Asociación Civil Impacta Salud y Educación, Lima, Peru; 3 Program in Global Health, University of California at Los Angeles, Los Angeles, CA, USA; 4 Asociación Civil Selva Amazónica, Iquitos, Peru; 5 Centro Medico Cayetano Heredia, Pucallpa, Peru; 6 U.S. Naval Medical Research Unit No. 6 (NAMRU-6), Callao, Peru

## Abstract

**Background:**

Knowledge of a sex partner’s HIV serostatus can influence sexual behavior and inform harm-reduction strategies. We sought to determine how often Peruvian men who have sex with men (MSM) and transgender women (TW) knew the HIV serostatus of their sex partners, if this knowledge was associated with any predictive factors or unprotected anal intercourse (UAI), and if UAI was associated with partner serostatus.

**Methods:**

We analyzed data from the 2008 Peruvian MSM Sentinel Surveillance Survey. Data were collected by CASI about each participant’s three most recent male sex partners. Primary outcome was knowledge of a partner's HIV test result. Multivariate analysis assessed the effect of age, education, sexual identity, number of male partners, alcohol use during intercourse, type of partnership and length of partnership using logistic regression.

**Results:**

735 participants provided data on 1,643 of their most recent sex partners from the last 3 months. 179/735 (24.4%) of all participants knew HIV test results for at least one of their 3 most recent partners, corresponding to 230/1643 (14.0%) of all sexual partnerships in the last 3 months. In multivariate analysis, casual (OR: 0.27, 95% CI: 0.17-0.42) and exchange sex (OR: 0.31, 95% CI: 0.11-0.88) partners, compared to stable partners, were negatively associated with knowledge of partner serostatus, whereas relationships lasting longer than one night (<3 months OR: 2.20, 95% CI: 1.39-3.51; 3 months to 1 year OR: 3.00, 95% CI: 1.80-5.01; ≥ 1 year OR: 4.13, 95% CI: 2.40-7.10) were positively associated with knowledge of partner serostatus. Knowledge of partner serostatus was not associated with unprotected anal intercourse with that partner.

**Conclusions:**

Few MSM and TW in Peru know their partners’ HIV serostatus. Our findings suggest that the type and length of partnership influence the likelihood of knowing a partner’s serostatus. Further research should explore the contexts and practices of partner communication, their effect on sexual behavior, and interventions to promote discussion of HIV testing and serostatus as an HIV prevention strategy in this population.

## Background

Knowledge of sex partner HIV serostatus can influence perception of sexual risk and may inform harm-reduction strategies for men who have sex with men (MSM) and transgendered women (TW) [1]. Currently little is known about partnership dynamics in Peruvian MSM and TW, including whether they discuss HIV testing, serostatus, or risk-reduction behaviors. In order to develop an initial understanding of communication about serostatus between Peruvian MSM and TW sex partners, we analyzed data from the 2008 HIV Sentinel Surveillance Survey of Peruvian MSM and TW to assess for factors associated with having knowledge of a sex partner’s HIV serostatus, and whether or not this knowledge or the partner’s serostatus itself is associated with unprotected anal intercourse (UAI) with that partner.

## Methods

### Study design

We conducted a secondary analysis of HIV surveillance data collected from April 2008 to September 2009 in three cities – Lima, Iquitos, and Pucallpa. Sentinel surveillance surveys have been regularly administered to high-risk MSM and TW in Peru since 1996 to assess emerging trends in the HIV and STI epidemics [2].

### Participant selection

Participants were recruited through “snowball” sampling techniques as well as by trained peer outreach workers who represented diverse MSM sub-cultures. Recruitment strategies included the use of posters, distribution of flyers, and informational meetings at previously-mapped venues in participating cities. Potential participants were referred by outreach workers to sentinel study sites where they were enrolled in the study. Participants were provided with condoms and reimbursement for the cost of transportation.

Enrollment was limited to anatomically born males at least 18 years old who reported anal sex with a male or male-to-female transgender partner in the past 6 months, who had not been tested for HIV in the previous 12 months, and who had never had a positive HIV test result, and who resided in one of the participating cities. Enrollment was also limited to subjects who reported at least one of the following high-risk sexual behaviors: insertive or receptive anal intercourse without a condom during the last sexual episode; insertive or receptive anal intercourse with more than 5 partners in the last 6 months; receipt of money, drugs, gifts, or accommodation in exchange for sex in the last 6 months; STI diagnosis in the last 6 months; or an HIV positive partner in the last 6 months.

Persons were excluded if they were HIV positive by self-report, previously enrolled in an HIV vaccine or clinical trial, or had a mental or psychiatric condition that prevented them from providing informed consent. Of the 899 participants who were enrolled in the surveillance study, our analysis excluded those who did not report a male sexual partner within the preceding 3 months. The final number of participants included in our analysis was 735.

### Data collection

All participants completed an anonymous survey with the use of CASI (Computer Assisted Self-Interviewing). The first part of the survey included sections on basic demographics, knowledge of HIV/STIs, history of STIs (past 6 months), access and frequency of use of condoms and lubricants, sexual role and identity, exchange sex activity (past 6 months), number of male and female partners (past 3 months), and alcohol and drug use (past month). The remaining sections asked an identical set of partnership and event-level questions about the last sexual encounter with each of the participant’s three most recent partners in the past 3 months.

The surveillance protocol received ethics approval for human subjects research from the Asociación Civil Impacta Salud y Educación (IMPACTA) Institutional Bioethics Committee and the U.S. Naval Medical Research Center Detachment (NMRCD, now Naval Medical Research Unit-6) Institutional Review Board (IRB). Secondary analysis of deidentified data was considered exempt from review by the Office of the Human Research Protection Program of the University of California, Los Angeles.

### Analysis

The primary outcome for our analysis was whether the participant knew their partner’s serostatus. If participants responded that their partner was HIV-positive or HIV-negative, they were categorized as having knowledge of their partner’s serostatus. If they reported that the partner had not been tested, or if the partner’s test result was not known, they were categorized as not having knowledge of their partner’s serostatus.

We developed a conceptual framework for our multivariate model prior to conducting the analysis. An extensive review of the literature and available data from the survey database guided our choice of predictor variables for our conceptual framework. As such, the final multivariate model included some variables which were not significantly associated with the primary outcome in bivariate analyses. Variables at both the participant level (age, education, sexual identity, number of male partners) and the partnership level (type and length of partnership, participant use of alcohol during sexual intercourse with the partner) were included, but many others, such as participating city, could not be included in the final multivariate model because of potential instability of the model. The inadequate sample size of TW precluded a separate analysis for this group.

Since the dependent variable - knowledge of partner HIV serostatus – was a partnership-level variable, and each participant reported data on up to three partnerships, data aggregation would violate the assumption of total independence between units under analysis (those units being partnerships, not participants). Therefore, bivariate and multivariate logistic regression analyses were conducted by clustering results to account for correlation of data. Units (partnerships) were clustered by using a variable that uniquely identified each participant. Crude and adjusted odds ratios (OR) are presented with 95% confidence intervals. We considered values statistically significant if the 95% confidence interval did not include 1.

We also analyzed the association between knowledge of a sex partner’s HIV serostatus and unprotected anal intercourse (UAI) with that partner as well as the association between the partner’s serostatus itself and the likelihood of unprotected anal intercourse . Sexual behavior at the last sexual encounter with each partner was dichotomized into unprotected anal intercourse (UAI) and non-UAI, which included anal intercourse with a condom as well as any non-penetrative forms of sex. Multivariate analysis using logistic regression clustered by participant adjusted for the effects of the same predictor variables. All data analyses were conducted using Stata 11.2 (College Station, TX).

## Results

### Study population

We analyzed data from 735 participants who reported at least one male partner in the last 3 months. These 735 high-risk MSM and TW provided data on 1,643 sex partnerships. Enrollment was evenly distributed across all cities.

### Participant characteristics

The median age of our study sample was 26 years (IQR: 22–33) with a range of 18 to 68 years. Participants reported a median of 5 male partners (IQR: 2–10) in the last three months, and 180 (24.5%) also reported having at least one female partner in the last 3 months. Additional demographic characteristics are reported in Table 1.

**Table 1:** **Characteristics of high**-**risk MSM in Lima**, **Pucallpa**, **and Iquitos**, **April 2008** – **September 2009**; **n**=**735**

	**Number (%)**
**Age** (**years**)	
<25	300 (40.9)
≥ 25	434 (59.1)
**Education level**^1^	
≤ Secondary	458 (62.3)
Superior/Technical	277 (37.7)
**Sexual identity**	
Heterosexual	33 (5.0)
Homosexual	355 (53.5)
Bisexual	164 (24.7)
Transgendered	111 (16.7)
**Sexual role** (**last 5 years**)	
Pasivo (Receptive)	380 (54.0)
Activo (Insertive)	221 (31.4)
Moderno (Versatile)	103 (14.6)
**Number of male partners**	
1	108 (15.5)
2-4	229 (33.0)
5-9	164 (23.6)
10 or more	194 (27.9)
**Number of female partners**	
0	555 (75.5)
1	55 (7.5)
2-4	88 (12.0)
5-9	27 (3.7)
10 or more	10 (1.4)
**Exchange sex activity** (**last 6 months**)	298 (40.9)
**Alcohol use** (**last month**)	648 (89.9)
**Drug use** (**last month**)	82 (11.3)

^1^Secondary education in Peru includes grades 7–11. Superior or technical education is considered to be any education obtained after high school (grade 11), including a professional university degree, or university or non-university technical degree.

### Partnership characteristics

Of the 1,643 partnerships reported, about one-fifth were described as stable. The median length of all partnerships was 4 days (IQR: 0–150 days). The median length for stable partnerships was 4.9 months (IQR: 0–24 months), and 0 days for both casual and exchange sex partnerships (IQR: 0–24 months and 0–3.5 months, respectively), i.e. the partnership was limited to a single encounter. Additional partnership characteristics are reported in Table 2.

**Table 2:** **Characteristics of male sex partnerships reported by high**-**risk MSM during past three months**; **n**=**1643**

	**Number (%)**
**Type of relationship**	
Stable	328 (20.3)
Casual^2^	1,189 (73.7)
Commercial sex client or worker	96 (6.0)
**Length of relationship**	
Single contact	727 (47.2)
Less than 3 months	322 (20.9)
3 months to less than 1 year	264 (17.1)
≥ 1 year	228 (14.8)
**Sexual role of partner**	
Pasivo (Receptive)	471 (29.2)
Activo (Insertive)	940 (58.4)
Moderno (Versatile)	200 (12.4)
**Alcohol use during last sexual encounter**	626 (40.2)
**Drug use during last sexual encounter**	180 (12.0)

^**2**^Sex partnerships described as “casual” by participants include those with both single and multiple sexual encounters.

### Knowledge of sex partner HIV serostatus

Among all participants, 24.4% (179/735) knew the serostatus for at least one of their three most recent sex partners, corresponding to 14.0% (230/1643) of all sexual partnerships reported in the previous 3 months. Of the 230 partners whose serostatus was known, 13 (5.7%) were reported as HIV-positive while 213 (94.3%) were reported as HIV-negative.

### Sexual behavior

67.3% of all participants had engaged in unprotected anal sex (UAI) with at least one partner at their last sexual encounter within the last 3 months. Participants reported UAI during roughly half (52.6%) of all reported partnerships during the last sexual encounter, and in 52.4% (741/1413) of partnerships where the partner’s HIV status was unknown.

### Factors predicting knowledge of sex partner HIV serostatus

In bivariate analyses, knowledge of partner HIV serostatus was positively associated with increasing length of the partnership, and negatively associated with increasing number of recent male partners as well as with casual or exchange sex types of relationships (Table 3).

**Table 3:** **Factors associated with knowledge of partner**’**s serostatus among last three sex partnerships**, **past three months**

	**OR (95% CI)**	**Adjusted OR (95% CI)**
**Age**		
<25	Ref	Ref
≥ 25	1.02 (0.72-1.44)	0.90 (0.60-1.36)
**Education level**		
≤ Secondary	Ref	Ref
Superior/Technical	1.34 (0.96-1.88)	1.15 (0.76-1.75)
**Sexual identity**		
Heterosexual	Ref	Ref
Bisexual	0.64 (0.28-1.42)	0.49 (0.17-1.35)
Homosexual	0.77 (0.34-1.78)	0.65 (0.23-1.83)
Transgendered	0.53 (0.22-1.30)	0.34 (0.11-1.03)
**Number of male partners**		
1	Ref	Ref
2-4	0.49 (0.28-0.84)*	0.68 (0.35-1.32)
5-9	0.57 (0.33-0.98)*	1.12 (0.58-2.17)
10 or more	0.42 (0.25-0.72)*	1.04 (0.55-1.95)
**Type of partnership**		
Stable	Ref	Ref
Casual	0.21 (0.15-0.30)*	0.27 (0.17-0.42)*
Commercial sex client or worker	0.19 (0.08-0.44)*	0.31 (0.11-0.88)*
**Length of partnership**		
Single contact	Ref	Ref
Less than 3 months	2.26 (1.50-3.40)*	2.20 (1.39-3.51)*
3 months to less than 1 year	3.01 (1.94-4.66)*	3.00 (1.80-5.01)*
≥ 1 year	5.52 (3.53-8.61)*	4.13 (2.40-7.10)*
**Alcohol use during last sexual encounter**		
No	Ref	Ref
Yes	0.81 (0.57-1.16)	1.34 (0.87-2.09)

* statistically significant based on 95% confidence interval that does not include 1.

After adjustment for age, education, sexual identity, number of male partners, and alchol use during the last sexual encounter, only the type and length of relationship were significantly associated with knowledge of partner serostatus (Table 3).

### Association of UAI with knowledge of partner serostatus and association of UAI with partner HIV serostatus

Knowledge of partner HIV serostatus was not significantly associated with likelihood of engaging in UAI with that partner in multivariate analysis. Participants with known HIV-positive partners [OR: 0.21; 95% CI: 0.04-1.03] or known HIV-negative partners [OR: 0.96; 95% CI: 0.63-1.46] appeared to be less likely to engage in UAI compared to those who did not know the serostatus of their sex partners, but these results were not statistically significant.

## Discussion

Knowledge of a partner’s HIV serostatus is uncommon among high-risk MSM and TW in Peru. The rate of knowledge of partner serostatus ranged from 8.3% for exchange sex partners to 32.6% for stable partners, much lower than that found in previous studies of MSM populations in the U.S. and Australia, which have reported knowledge of partner serostatus ranging from 15.6% for casual partners [3] to 86.3% for stable partners [4]. Of partners whose serostatus was known, only 5.7% were reported HIV-positive, a level far below the actual HIV prevalence rate of 9.6-30.0% among MSM and TW in Peru [2,5-8]. These results suggest that some MSM and TW are unaware that one or more of their partners is HIV-positive. The implication of HIV-positive persons reporting their HIV status to their sex partners as negative or unknown, whether intentional or not, is that the ability of sex partners to effectively utilize seroadaptive and self-protective behaviors may be diminished.

Knowledge of partner serostatus was dependent on both the type and length of partnership in our multivariate analysis. Our results indicate that even among partners qualitatively categorized as casual, those who have known each other for a longer period of time were more likely to share information about HIV serostatus. At the same time, some long-term partners may be considered “casual” and therefore less likely to communicate about HIV serostatus than “stable” partners who have known each other for a shorter period of time. Previous research on partner notification in high-risk Peruvian men and women has similarly demonstrated a decreased likelihood to notify casual as compared to stable partners after being recently diagnosed with HIV [9]. HIV-related stigma underlies accounts of “fear” and “embarrasment” and is a commonly reported reason for failure to discuss HIV with MSM partners [10-13]. A greater sense of intimacy, trust and/or mutual reponsibility found in stable and longer-term partnerships may help to overcome stigma and promote communication about serostatus.

Exchange sex partnerships were also associated with a decreased likelihood of knowledge of partner serostatus. Exchange sex in Peru often occurs in low-income areas where the social and economic vulnerabilities of both homosexual and marginalized heterosexual men can result in a power differential which hinders communication and negotiation of sexual risk [14]. Future prevention research may include behavioral interventions with a focus on building communication skills, offering social support, and emphasizing the importance of initiating discussions about HIV testing, serostatus, and safer sex, specifically with casual or new partners and during exchange sex [15].

Neither knowledge of a partner’s HIV test result nor the serostatus itself was significantly associated with UAI with that partner. Although results seemed to indicate a pattern of protective behavior with HIV-positive partners, the association was not statistically significant. The lack of association with partner serostatus may have been due to insufficient power since this part of the analysis could only be conducted with the 230 partners whose serostatus was known. The lack of association between unprotected intercourse and knowledge of partner serostatus corroborates results from a study of Latino gay men in the U.S. which also found no association between knowledge of partner serostatus and likelihood of engaging in UAI [16]. Knowledge of partner serostatus alone may not be sufficient to prompt a change in sexual behavior; condom use or safer sex strategies may need to be discussed in addition to sharing serostatus in order to increase the rate of protected sex [16-18].

There are a number of limitations in our study. First, all data were self-reported and therefore subject to recall and social desirability bias. Second, this study recruited a convenience-based sample of high-risk MSM in three cities and may not represent the Peruvian MSM and TW population as a whole. Although large samples representing a diverse range of MSM subcultures were included, men who participated in this study consisted mainly of those who visited socialization venues where study recruitment was conducted and who voluntarily accepted participation. MSM at higher risk for HIV and/or STI acquisition may have been more willing to participate or selected for in this survey based on eligibility criteria.

Other major limitations of our study may be attributed to its design as a secondary analysis. The outcome measure was based on a single question asking for the partner’s most recent HIV test result and was not suited to adequately evaluate the complex dynamics of partner communication about HIV testing and serostatus. It is likely that some of the participants had inaccurate or uncertain knowledge of their partner’s serostatus [19], and the veracity of the partner’s testing history and serostatus could not be determined without directly validating this information with each partner. Future studies should ask specific questions about what Peruvian MSM and TW discuss with their partners regarding HIV and safer sex, how often, when in relation sexual intercourse, where, who initiates, and how verbal discussion is initiated. Furthermore, it would be ideal to evaluate communication bi-directionally, asking not only what the participant knows about his partner, but also if he shared any information about his own sexual or HIV history with this partner.

## Conclusions

Our analysis adds to the literature on communication about HIV status among MSM and TW, and also highlights the need for further research on this topic in Peru. The lack of adequate data on the accuracy and use of personally disclosed information regarding partner HIV serostatus mandates caution in the use of serodisclosure or serosorting as an isolated prevention measure. Further research should explore the contexts and practices of partner communication, their effect on sexual behavior, and interventions to promote discussion of HIV testing and serostatus as an HIV prevention strategy among MSM and TW.

## Competing interests

The authors declare that they have no competing financial interests or conflicts of interest.

## Authors’ contributions

SN conceived and designed the secondary analysis, performed the statistical analysis and drafted the manuscript. ES participated in the conception and design of the secondary analysis, performed the statistical analysis, and critically revised the manuscript. JP was involved in the design and implementation of the original surveillance study, participated in the design of the secondary analysis, created the original database, and participated in the statistical analysis. KAK participated in the statistical analysis and critically revised the manuscript. PS, MC, and AO were all involved in the implementation of and and acquisition of data from the 2008 Sentinel Surveillance protocol in Lima, Iquitos, and Pucallpa, respectively. JC participated in the conception and design of the secondary analysis and critically revised the manuscript. JS was involved in the design and implementation of the original surveillance study, participated in the design of the secondary analysis and critically revised the manuscript. JRL was involved in the design and implementation of the original surveillance study, participated in the design of the secondary analysis and critically revised the manuscript. All authors contributed to the interpretation of the data and the preparation of the manuscript.

## Disclaimers

The study protocol and written informed consents were approved by the Asociación Civil Impacta Salud y Educación Institutional Bioethics Committee and U.S. Naval Medical Research Unit Six (NAMRU-6) Institutional Review Board in compliance with all applicable federal regulations governing the protection of human subjects.

Silvia M. Montano is an employee of the U.S. Government. This work was prepared as part of their official duties. Title 17 U.S.C. 105 provides that Copyright protection under this title is not available for any work of the U.S. Government. Title 17 U.S.C. 101 defines a U.S. Government work as a work prepared by a military service member or employee of the U.S. Government as part of that person's official duties.

The opinions and assertions contained in this manuscript are those of the authors and do not necessarily reflect the official policy or position of the U.S. Department of the Navy, U.S. Department of Defense, the U.S. Government or of any of the other organizations listed.

## Pre-publication history

The pre-publication history for this paper can be accessed here:

http://www.biomedcentral.com/1471-2458/13/181/prepub
